# Whole-transcriptome analysis of rat cavernosum and identification of circRNA-miRNA-mRNA networks to investigate nerve injury erectile dysfunction pathogenesis

**DOI:** 10.1080/21655979.2021.1973863

**Published:** 2021-09-14

**Authors:** Jie Huang, Jianxiong Ma, Jie Wang, Ke Ma, Kang Zhou, Wenjie Huang, Fan Zhao, Bodong Lv, Qing Hu

**Affiliations:** a The Second Clinical Medical College, Zhejiang Chinese Medical University, Hangzhou, China; b Department of Urology, First People’s Hospital of Yuhang District, Hangzhou, China; c Department of Urology, Second Affiliated Hospital, Zhejiang University School of Medicine, Hangzhou, China; d Department of Urology and Andrology, The Second Affiliated Hospital of Zhejiang Chinese Medical University, Hangzhou, China; e Department of Urology, Affiliated Hospital of Nantong University, Nantong, Jiangsu, China; f Zhejiang Provincial Key Laboratory of Prevention and Treatment of Combined Chinese and Western Sexual Dysfunction, Zhejiang Chinese Medical University, Hangzhou, China; g Department of Urology, The Second Affiliated Hospital of Zhejiang University, Hangzhou, China

**Keywords:** Erectile dysfunction, circular RNA, ceRNA network, bioinformatics analysis, energy metabolism

## Abstract

There is growing evidence that circular RNAs (circRNAs) play a vital role in many kinds of diseases, including erectile dysfunction (ED). Nevertheless, the role of circRNAs in cavernous nerve-damaging ED (CNI-ED) is unknown. Here, we aimed to discover novel circRNAs, probed their potential role in the CNI-ED, and construct a ceRNA network of circRNAs. Twelve male Sprague Dawley rats were randomly divided into 2 groups by us: bilateral cavernous nerve crush (BCNC) and control groups. Four weeks after surgery, the spongy smooth muscle tissue of the rat penis was sequenced using high-throughput full transcriptome sequencing. We analyzed the expression of circRNAs, miRNAs, and mRNAs in the two groups. Twenty circRNAs with significantly different expressions were selected for RT-qPCR. CeRNA network of circRNAs was established using Cytoscape. GO and KEGG analysis was done by R package. Sequencing showed that 4,587 circRNAs, 762 miRNAs, and 21,661 mRNAs were dysregulated in the BCNC group. The top 20 differentially expressed circRNAs were further verified via RT-qPCR. The ceRNA network contained ten circRNAs, six miRNAs, and 227 mRNAs, including 23 circRNA-miRNA pairs and 227 miRNA-mRNA pairs. GO and KEGG analysis suggested that these ten circRNAs could main regulate energy metabolism processes. A protein‐protein interaction network was constructed with the mRNAs in ceRNA network, and five hub genes were identified. Our study revealed a potential link between circRNAs, miRNAs, and mRNAs in CNI-ED, suggesting that circRNAs may contribute to the occurrence of ED by regulating the cellular energy metabolism in CNI-ED.

## Introduction

Prostate cancer is one of the most common malignancy in men worldwide. Recent studies indicate that prostate cancer makes up more than one in five new cancer cases is in the United States [1]. Radical prostatectomy (Rp) is the major treatment modality to reduce cancer mortality in patients with localized prostate cancer [2]. Unfortunately, due to the location of the prostate, men receiving RP treatment may suffer side effects, which can have a long-term impact. Even though various neurosparing methods have been promoted in recent years, the incidence of erectile dysfunction (ED) after RP is still high at about 11–87% [3,4]. The main cause of ED is surgical damage to the cavernosal nerve in patients with prostate cancer. After nerve injury, the nerve control of the cavernous body of the penis is lost, and a variety of pathological and physiological changes occur, such as smooth muscle diastolic dysfunction of the cavernous body of the penis and the occurrence of penile fibrosis, which lead to conventional treatment such as oral PDE5 inhibitors, vacuum negative pressure suction, and local injection of vasoactive drugs into the cavernous bod have poor efficacy, so ED after RP is still a long-term problem for most patients [2,5–7]. Therefore, finding the underlying mechanism is very critical for the treatment of post-RP ED.

Circular RNAs (circRNAs) are evolutionarily conserved transcripts, making up a mass class of non-coding RNAs, which are produced by backsplicing [8]. For the past few years, mounting evidence has demonstrated the critical role of circRNAs in biological processes by modulating protein function, acting as miRNA or protein inhibitors (sponges), or by encoding proteins themselves [9–11]. In addition, circRNAs are closely related a variety of diseases, such as diabetes mellitus and its complications, cardiovascular disease, neurological disorders, osteoporosis and cancers [12–14]. Recently, circRNAs as miRNA sponges regulating biological functions have been gradually understood, and a large number of studies have shown that miRNAs play a vital role in the pathogenesis and treatment of ED [15,16]. However, how circRNAs regulate miRNAs and ultimately regulate cavernous nerve injury erectile dysfunction (CNI-ED) remains unknown [17].

**Figure 1.**
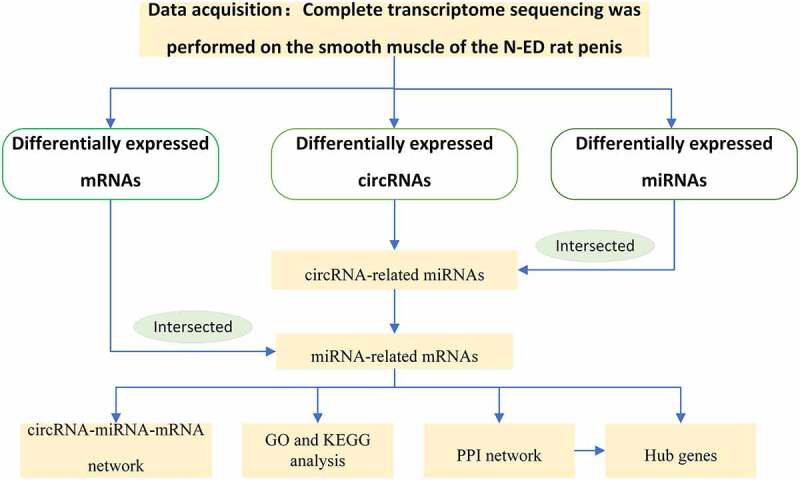
The process of constructing circular RNA ceRNA network and looking for hub genes in CNI-ED

The above research suggested one possibility that a comprehensive study of the function of competing endogenous RNA (ceRNA) network of circRNAs and the ability of circRNA to encode proteins is an effective strategy for understanding CNI-ED. In this study, we designed to build a ceRNA network of circRNAs and discover the potential of circRNAs to encode proteins. Illumination of the potential link between CNI-ED and ceRNA network of circRNAs could suggest new strategies for treating this disease. The study was designed to identify the differentially expressed circRNAs and miRNAs and mRNAs by whole-transcriptome sequencing of CNI-ED and normal rats. A ceRNA network was subsequently constructed to reveal the potential role of circRNAs in CNI-ED. We found that circRNAs lead to CNI-ED by regulating energy metabolism, in which *Ccna2, Cxcl10, Pld1, Mapk11, and Mboat2* may play an important role.The workflow of the our current research is shown in Figure 1.

## Materials and methods

### Animals

Experimental animal ethics was approved by the Laboratory Animal Center of Zhejiang University of Traditional Chinese Medicine. All rats were raised in the Experimental Animal Center of Zhejiang Chinese Medicine University in a special sterile environment: water and food were freely available, and a 12-hour cycle of day and night and maintained at 20–24°C. Twelve male SD rats weighed between 300 and 350 g and were 8 weeks old (SIPPER-BK Laboratory Animals, Shanghai, China) were randomly divided into experimental group and control group and done as follows. All surgical procedures and operations were followed the animal ethics guidelines of the National Health and Research Institutes. The rat were anesthetized with 3% pentobarbital sodium (1.5 × 10^−5^L/100 g). In the model group (n = 6), only bilateral cavernosal nerves(CN) were isolated but not damaged. In the bilateral cavernous nerve crush injury (BCNC) group (n = 6), the damaged bilateral cavernous nerves of rats were pinched with vascular forceps twice for 60 seconds [18,19].

### Collection of sequencing tissues

28 days after the operation, utilizing 3% pentobarbital sodium (1.5 × 10^−5^L/100 g) anesthesia in rats, after waiting for anesthesia effect, carefully separate the rat penis, then remove the penis sponge tissue, rinsing with pre-cooled PBS liquid, carefully remove other tissues from the penis. The rats were then euthanized in carbon dioxide. Finally, all the sponge tissue after marking, placed in the −80°C refrigerator for further histologic studies.

### RNA library construction and sequencing

Transcriptome sequencing was performed on 3 normal group rats and 3 model group rats. The whole transcriptome sequencing work was completed by Hangzhou Lianchuan Biotechnology Co., LTD. The specific operations are as follows: total RNA in the cavernous tissue of the rat was isolated and purified using Trizol reagent (Invitrogen, Carlsbad, CA, USA). The RNA concentration and purity we extracted were tested by using NanoDrop ND-1000 (NanoDrop, Wilmington, DE, USA). The RNA integrity was assessed by Agilent 2100 with RIN number >7.0. The ribosomal RNA was depleted by approximately 5ug of total RNA through the Ribo-Zero™ rRNA Removal Kit (Illumina, San Diego, USA). When eliminating ribosomal RNAs the left RNAs were sliced up at high temperatures. The cleaved RNAs were then reverse transcribed into cDNA, which were labeled with U-labeled to synthesize 2-strand DNA. An A-base was added to the blunt ends of each strand, preparing them for ligation to the indexed adapters. Single-or dual-index adapters are ligated to the fragments, and size selection was performed with AMPureXP beads. The two strands of DNA labeled by U were treated with the refractory UDG enzyme for PCR operation under the following operating conditions: 95°C for 3 min; 8 cycles at 98°C for 15 seconds, 60°C for 15 sec, and 72°C for 30 seconds; and then 72°C for 5 min. The final PCR cDNA library was about 250–350 bp in size. In the end, The paired-end sequencing was performed by us according to the protocol from Illumina Hiseq 4000 (LC Bio, China) [20].

### RNA extraction and RT-qPCR validation

Twenty significantly different circrnas were selected for qPCR validation. Total RNA was extracted using the TRlzol method. RT-qPCR primers (Table 1) were designed by primer 5.0. The RNA concentration and purity we extracted were tested by using NanoDrop ND-1000 (NanoDrop, Wilmington, DE, USA). The RNA integrity was assessed by Agilent 2100 with RIN number >7.0. The OD260/280 absorbance ratios of all samples ranged from 1.8 to 2.0. Reverse transcription of cDNA was completed with the HiScript II Q Select RT SuperMix for qPCR (R233) (Vazyme, China), and the qPCR procedure was performed according to the StepOne Plus Real-Time PCR system (Applied Biosystems, USA). GADPH was used as an endogenous control to normalize each sample. PCR amplifification effificiencies were established by means of calibration curves.

**Table 1.** The circRNA primer sequence

circRNAname	Forward Primer 5'-3'	Reverse Primer 5'-3'
circRNA2986	GTGAACCTTGTGTGAGCAGC	CTTACCGTCTGTGGGCAGTT
circRNA3009	GAGGCATCCAAGAGGTCA	AGGGTCCATTTGGTGTCC
circRNA2982	GCTGCCCAAGACTTCTGA	AATCCTTTCTGCCTGCTT
ciRNA167	CCGTGGTTCAGGAGAT	GTCAGGCGACTGTAGGT
ciRNA85	GTCGTACAAGCCATCTCA	CTCCCAAGTTCAACATTAA
ciRNA351	GCCAGTGTCTGGGAAACTCTT	GAAAGGCACTACAACCAGGG
circRNA945	AGGCAGATGATGGATAGCACA	ACTGTTGGATCAGGATGGCAG
circRNA906	CTCTTGGCAGTGGGTAAA	AACAGAGCAACACGAGCC
circRNA2760	TGTCAGCCCAGGTCTATT	CCAGCCACAGTGCTCATT
circRNA2984	TAGAATGATGCTGGCTGAA	GTCTGATGGATTCCTGGTC
circRNA1002	TGTCAGCCCAGGTCTATT	CCAGCCACAGTGCTCATT
ciRNA109	TACTTTGCGGCTGTTCTGA	CATGCGCTATGCTTCTACC
circRNA2983	TGGATTCGCTTCCTCCTTTGT	TTGAGAAGTGCTTGCAGTGGT
ciRNA171	GAACTGTGGACACCTGTTGC	ATTCCCGTGTTGGAGTTGGT
circRNA2981	TGAACCTCGTCAATACCT	CTTCCCGTAGAATAACAAA
circRNA1687	CCTTGGGCAGATGGTTTA	R: 5GGAGGAGCCTGGATTTCT
circRNA1022	AAGTTAGGCAGCCAGCAC	CGAGTATGGAAGCGAAGG
circRNA893	TCCTGCTTGGCGATGAGT	CATCCCTTCTGGGCACCT
circRNA1056	GCCATTGACTGCTCTGTA	CTTGGTCTGAAAGGGAAC
circRNA1063	ACATTGCTGATGGTGTATTT	ATCCAGTTCCTTTAAGTGAG
GAPDH	TGGACCTGACCTGCCGTCTA	CTGCTTCACCACCTTCTTGA

### Construction of circRNA-miRNA-mRNA ceRNA network

The differential expression between the normal and model groups was further analyzed using the lima package. The DE circRNAs and mRNAs were screened according to the following criteria: Log2 (fold change) > 1 and P ≤ 0.05 [21], The miRNAs screening was as follows: P ≤ 0.1. Utilizing the Circular RNA Interactome (http://circinteractome.nia.nih.gov) database to predict miRNAs that circular RNA may play a spongy role. We obtained what we needed miRNAs by intersecting the meaningfully differentially expressed miRNAs(DEmiRNAs) with the predicted miRNAs. The downstream target genes of miRNA were predicted from the miRanda and TargetScan. Differentially expressed mRNAs (DEmRs) were obtained by intersecting predicted mRNAs and sequenced mRNAs. Finally, the network was constructed by Cytoscape 3.71.

### Gene Ontology (GO) and Kyoto Encyclopedia of Genes and Genomes (KEGG) analyses

CircRNAs act as miRNA sponges because of containing corresponding miRNA binding sites [9]. Translation of mRNA was inhibited by miRNA binding to the 3ʹ non-coding region of mRNAs [22]. Subsequently, ceRNA networks with significantly differentially expressed circRNAs were constructed by us. Moreover, We used Cytoscape 3.7.1 (http://cytoscape.org/) and the edgeR package to visualize the network. Then, we performed functional enrichment analysis of RNAs in the circular RNAs network. Gene Ontology (GO) and Kyoto Encyclopedia of Genes and Genomes (KEGG) analyses was performed on the online analysis platform of Lianchuan Biotechnology Co., Ltd(https://www.omicstudio.cn/). GO and KEGG analyses were performed using the ClusterProfler package that identified biological processes and pathways. Subsequently we used ClueGO to analyze the path correlation of the gene enrichment pathways in the ceRNA network. We used STRING to analyze mRNAs in the network and establish a protein-protein interaction (PPI) network to look for potential connections. Finally, CircAtlas 2.0 was used to predict circRNA conservation and protein-coding ability 22.

## Statistical analysis

SPSS 17.0 statistical software was used for data analysis. T test was used for measurement data between two groups. A value of P < 0.05 indicated that the difference was statistically significant, P > 0.05 indicates that the differences are not statistically significant.

## Results

In this study, we aimed to identify the differentially expressed circRNAs, miRNAs, and mRNAs by sequencing the CNI-ED rats and normal rats, and then constructed a ceRNA network centered on circRNAs. Through GO and KEGG analyses of related genes in the ceRNA network revealed that circRNAs regulate CNI-ED through energy metabolism process, suggesting that circRNAs can serve as treatment targets for CNI-ED.

### RNA transcriptome sequencing results

**Figure 2.**
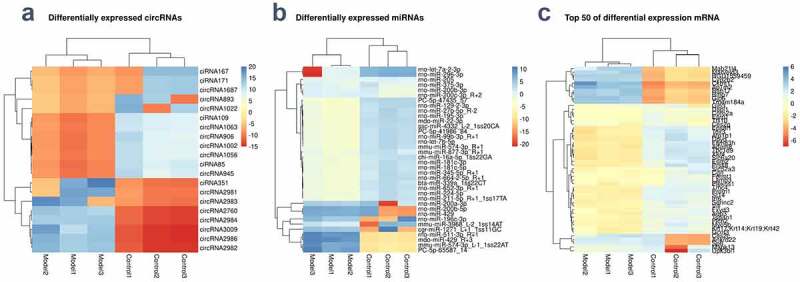
**Differential expression of RNAs** (a-c) The differences of circRNAs, miRNAs, and mRNA between the BNCI group and the normal control group were shown by heat map. High expression is shown in red and low expression in green

RNA transcriptome sequencing showed that 4,587 circRNAs, 762 miRNAs, and 21,661 mRNAs were dysregulated in the BCNC group. We also screened 20 differentially expressed circRNAs (eight upregulated and twelve downregulated circRNAs) and 1,908 DEmRNAs among which 1605 were up-regulated and 303 were down-regulated in rats; 36 DEmiRNAs (24 up-regulated and 12 down-regulated miRNAs) with P ≤ 0.1. Figure 2a, b, and c showed heat maps of DEcircRNAs, DEmiRNAs, and DEmRNAs between the model group and the control group. Supplementary figure 1a, b, c showed a volcanic map of DE genes.

### Validation of DEcircRNA by RT-qPCR

**Figure 3.**
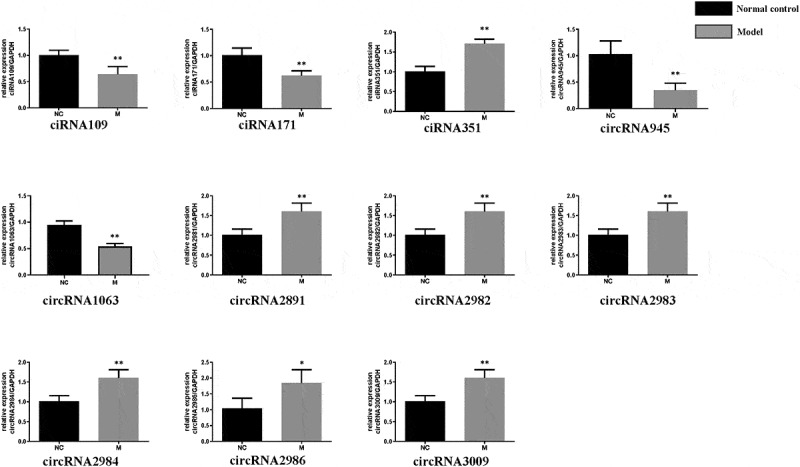
**Validation of circRNA by RT-qPCR**: Relative expression levels of differentially expressed circRNAs in model group and normal group. Mean ± SD, n = 3; The two-tailed Student t-test was used to assess statistical significance: *P < 0.05 and **P < 0.01 represent differential expression between the model group and normal group

To further ascertain circRNAs associated with CNI-ED, the 20 dysregulated circRNA were chosen for validation using RT-qPCR. As shown in Figure 3, several differentially expressed circRNAs were identified via RT-qPCR, including ciRNA351, circRNA2981, circRNA2982, circRNA2983, circRNA2984, circRNA2986, circRNA3009 were further overexpressed in the model group. On the contrary, circRNA945, circRNA1063, ciRNA109, ciRNA171 were downregulated in the BCNC group. However, the remaining nine circRNAs did not show significant differential expression, detected using RT-qPCR.

### Prediction of interactions between circRNAs, miRNAs, and mRNAs

**Figure 4.**
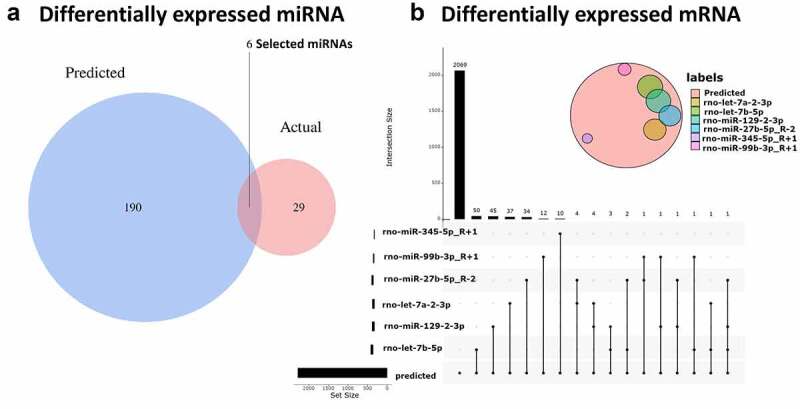
**The connections between circRNA, miRNA and mRNA** (a) Blue represents miRNAs that are capable of spongy circRNAs predicted by CircInteractome, red represents the differential miRNAs obtained by RNA sequencing (p ≤ 0.1), and the overlaps represent the differentially expressed miRNAs that we ultimately need. (b) The red circle represents the predicted mRNA that can target all miRNAs, and the other colored circles represent the intersection of each miRNA’s predicted and actual sequencing results. Finally, we obtained 50 target genes of rno-let-7b-5p, 45 target genes of rno-miR-129-2-3p, 37 target genes of rno-let-7a-2-3p, 34 target genes of rno-miR-27b-5p_R-2, 12 target genes of rno-miR-99b-3p_R + 1, and 12 target genes of rno-miR-345-5p_R + 1

To determine whether the previously selected differential circRNAs play a similar role in CNI-ED and predict their target miRNAs, the online database CircInteractome was used. We then obtained overlapping miRNAsby intersecting predicted and detected miRNAs (Figure 4a). Ultimately, 23 circRNA‐miRNA interactions were established, including ten circRNAs (seven upregulated and three downregulated) and six miRNAs (five upregulated and one downregulated miRNAs). TargetScan and miRWalk 3.0 were used to predict the mRNAs that miRNA might bind to, and the overlapping mRNAs were obtained by intersecting these DEmRNAs. These 227 mRNAs (Figure 4b) may play an important role in CNI-ED.

### Construction of ceRNA networks of circRNAs

**Figure 5.**
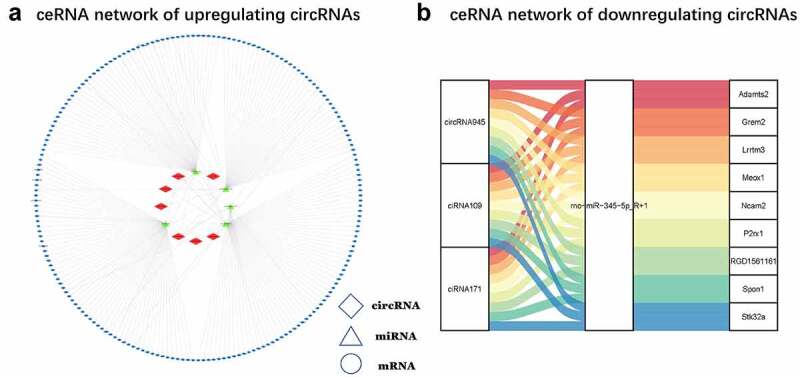
**ceRNA network** (a) Upregulated circRNA ceRNA network. The red diamonds stand for upregulated circRNAs, the green triangles represent down-regulated miRNAs, and the blue ovals denote upregulated miRNAs. (b) Down-regulated circRNA ceRNA networks in CNI-ED

In this study, we established a ceRNA networks of circRNAs showing the upregulated (Figure 5a) and downregulated (Figure 5b) circRNAs, to reveal the underlying relationship between between circRNAs, miRNAs, and mRNAs. This network contained ten circRNAs, six miRNAs, and 227 mRNAs, including 23 circRNA-miRNA pairs and 227 miRNA-mRNA pairs.

### Go and KEGG analysis of mRNAs in the ceRNA network

**Figure 6.**
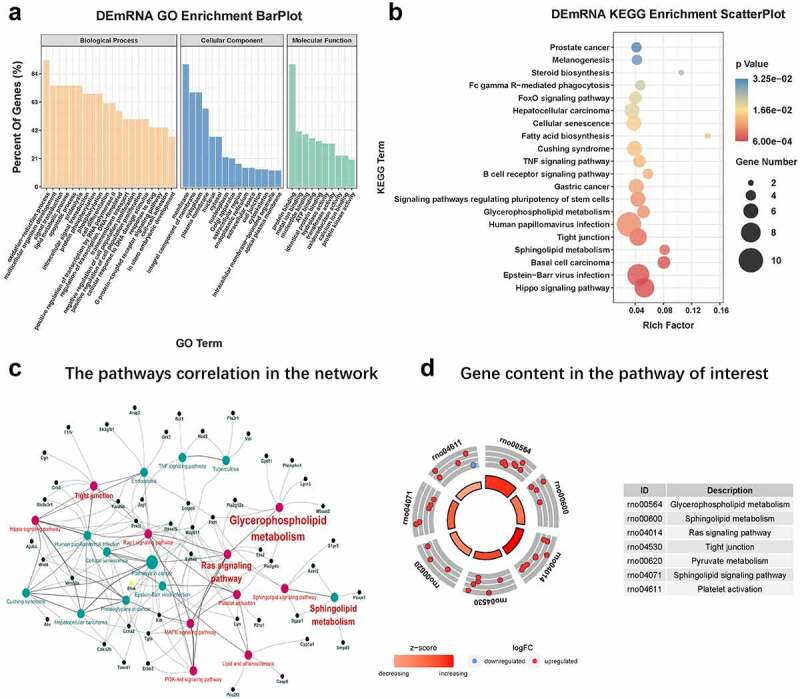
**GO and KEGG analysis** (a) The top 20, 15, and ten most meaningful GO terms in BP, CC, and MF in CNI-ED. orange bars represent MF terms. Blue bars represent CC terms. Light green bars represent BP. (b) Pathway prediction of KEGG analysis. The larger the circle, the greater the number of enriched genes. The redder the color, the higher the significance of enrichment pathway. (c) The pathways correlation in the ceRNA network. Red circles represent pathways associated with energy metabolism, and the black circles represent the genes associated with these pathways. (d) The number of genes contained in each pathway of interest. A circle represents a gene, red up and blue down

We performed KEGG and GO analysis of 227 DEmRNAs obtained. The top 20, 15, and ten most meaningful GO terms in biological processes(BP), cellular component(CC), and molecular function(MF) are shown in Figure 6a. GO analysis results showed that DEmRNAs mainly participated in oxidation-reduction processes, multicellular organism development, signal transduction, lipid metabolis, apoptosis, proteolysis, intracellular signal transduction, protein binding, metal ion binding, ATP binding.

According to the KEGG analysis, the main KEGG categories included genetic information processing metabolism, hippo signaling pathway (ID:rno04390), glycerophospholipid metabolism (ID: rno00564), sphingolipid metabolism (ID: rno00600), tight junctions (ID: rno04530), signaling pathways regulating stem cell pluripotency (ID:rno04550), fatty acid biosynthesis (ID:rno00061), and cellular senescence (ID:rno04218), which were significantly (p ≤ 0.01) enriched (Figure 6b). The pathways correlation in the network is shown in Figure 6c. The number of genes contained in each pathway of interest are shown in Figure 6d.

### PPI network analysis of mRNAs in ceRNA network

**Figure 7.**
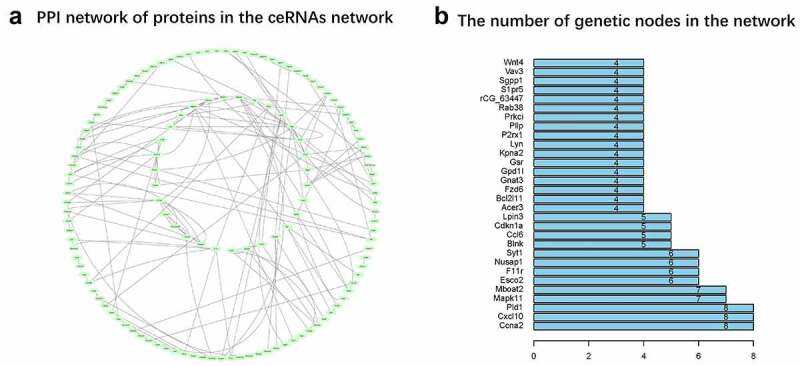
**PPI network** (a) The network is generated by String and the graph is generated by Cytoscope. The pale green oval represents an mRNA, and the lines represent a connection between the two mRNAs. (b) Nodes rank top 30 mRNAs in the network

A total of 134 mRNAs were found to interact and 332 protein pairs were included in the PPI network (Figure 7a). The number of relative pairs per protein are shown in Figure 7b. The results suggested that Ccna2, Cxcl10, Pld1, Mapk11, and Mboat2 might be the core genes of the regulatory network.

### The ability of DE circRNAs encoding protein and the conservation of DE circRNAs

Significant differentially expressed circRNAs were screened using circRNADb [23], revealing 12 circRNAs with internal ribosomal entry sites and open reading frames, suggesting that they might code proteins, and we also found that three circRNAs were conserved in humans and rats (Table 2).

**Table 2.** The ability of DE circRNAs encoding protein and the conservation

circRNA name	strand	chr	start	end	regulation	IREScount	ORFcount	conservation
circRNA2986	+	chr1	203279889	203317455	up	0	0	NO
circRNA3009	-	chr7	143274109	143324536	up	Not found in the website		
circRNA2982	-	X	119208336	119208618	up	4	2	NO
ciRNA167	-	chr8	93334030	93350656	down	10	7	NO
ciRNA85	-	chr11	46270461	46271069	down	10	3	NO
ciRNA351	-	chr16	79659797	79667893	up	1	4	NO
circRNA945	+	chr17	19160994	19225009	down	0	0	NO
circRNA906	+	chr1	99510577	99533851	down	Not found in the website		
circRNA2760	-	chr4	8223840	8242710	up	7	8	YES
circRNA2984	-	X	137706330	137733342	up	Not found in the website		
circRNA1002	-	chr4	8223841	8242710	down	7	8	YES
ciRNA109	-	chr13	107733302	107772361	down	9	10	NO
circRNA2983	-	X	119208336	119213196	up	0	0	NO
ciRNA171	-	chr20	4789931	4893922	down	Not found in the website		
circRNA2981	+	X	109996163	110063748	up	0	0	NO
circRNA1687	+	chr8	99640888	99717000	down	14	8	NO
circRNA1022	-	chr10	82760353	82760613	down	1	1	NO
circRNA893	+	chr8	130762666	130768360	down	13	8	NO
circRNA1056	+	chr7	26522315	26526603	down	6	6	NO
circRNA1063	-	chr2	206956638	206976810	down	2	15	YES

## Discussion

For the current study, we aimed to explore the roles of circRNAs of CNI-ED. Therefore, the expression level of RNA in CNI-ED was determined by second-generation high-throughput sequencing. Whole transcriptome sequencing showed that 4,587 circRNAs, 762 miRNA, and 21,661 mRNAs were dysregulated in the BCNC group. Twenty circRNAs with significant differences were verified by PT-qPCR. We identified eleven circRNAs that were significantly different between the normal control group and the model group by qPCR. Finally, circRNA945, ciRNA109, ciRNA171, circRNA2981, circRNA2984, circRNA2986, circRNA3009, ciRNA351, circRNA2982, and circRNA2983 were selected to construct the ceRNA network of circRNAs to predict their functions.

ED is the most common long-term complication after RP [4]. Currently, PDE-5 inhibitors are mainly used in the treatment of CNI-ED, but clinical studies have shown that sildenafil is not efficient in the treatment of CNI-ED, being effective in only 35% of patients [24]. The possible reason is that a series of complex pathophyseologic changes such as cavernosum fibrosis and cavernosum smooth muscle atrophy occurred after the loss of nerve control in the penis [2,5–7]. Therefore, it is of great practical significance to find a new treatment regime for CNI-ED.

Erectile function is regulated by the diastolic and contractile functions of corpus cavernous smooth muscle cells (CCSMCs). After the loss of innervation of the cavernous body in patients with CNI-ED, CCSMCs gradually deteriorate, mainly manifested as increased intracellular muscle protein degradation, significantly reduced muscle content, inhibited cell proliferation, and apoptosis [25–27], which eventually leads to ED.

CircRNAs are a new kind of endogenous non-coding RNAs, produced via head-to-tail backsplicing of one or more exons [11]. There is growing evidence that circRNAs play an important role in biological functions by acting as miRNA sponges [10]. A growing body of research shows that circRNAs are rich in species, stable in structure and conserved in sequence. Also, an increasing number of reports have shown that circRNAs affected RNA translation and expression by competitively binding miRNAs. CircRNAs can also regulate mRNA expression. In recent years, with the development of second-generation high-throughput RNA sequencing technology, many new circRNAs and their new functions have been discovered, such as adsorbing and regulating miRNA activity as miRNA sponges, binding to transcriptional regulatory elements or interacting with proteins to regulate gene transcription and translation [28–30]. For example, circRNA-HRCR regulates the progression of heart failure by targeting miRNA-233 [31], and the down-regulation of circRNA-DMNT3B leads to the occurrence of diabetic eye disease by targeting miRNA-20b-5p [14]. In addition, Guo et al. found the protective effect of RNA3503 on osteoarthritis by overexpressing the circRNA3503 in the extracellular vesicles of synovival mesmesymal stem cells [32].Existing research has also shown that circRNAs play a crucial role in muscle atrophy [33]; however, direct evidence about circRNAs in CNI-ED is still absent.

We performed GO analysis for mRNAs in the network, and the results showed that they were enriched mainly in oxidation-reduction processes, multicellular organism development, signal transduction, lipid metabolism, apoptosis, and proteolysis. These results suggested that energy metabolism, proteolysis, and apoptosis play important roles in the network of circRNAs regulating CNI-ED. Furthermore, KEGG pathway enrichment analysis showed that the mRNA in the ceRNA network of circRNAs was mainly concentrated in the hippo signaling pathway, glycerophospholipid metabolism, sphingolipid metabolism, tight junctions, signaling pathways regulating stem cell pluripotency, fatty acid biosynthesis, and cellular senescence. After cavernous nerve injury, a series of pathophysiological reactions, such as penile ischemia and hypoxia, cell phenotype transformation, increased apoptosis, and cavernous tissue fibrosis occur gradually due to the weakened stimulation of the muscle [5]. Furthermore, circRNAs may cause abnormalities in the energy metabolism through miRNA sponging and eventually lead to the occurrence of ED. This could be a potential treatment for CNI-ED.

Abnormal energy metabolism is closely related to the occurrence of various diseases. Mitochondria are the energy factories of cells, but their role in ED nerve injury is still unclear. We previously found that the PI3K/Akt pathway is affected in CCSMCs induced by hypoxia [34]. Hence, whether circRNAs affect the mitochondrial function in CCSMCs through the regulation of PI3K/Akt pathway and lead to the occurrence of ED still warrants further research.

*Cxcl10* is one of the hub genes in the ceRNA network of circRNAs established here, and KEGG analysis showed that *Cxcl10* regulates a variety of energy metabolism processes, such as glycerophospholipid metabolism, Ras signaling pathway, etc. Interestingly, we found that *Cxcl10* induces mitochondrial dysfunction of pancreatic cells, then leads to energy metabolism disorders, finally leading to apoptosis [35]. Recent studies have shown that miRNA-27b-5p targets *Cxcl10* to regulate its effects [36]. Another study suggested that miRNA-27b regulates mitochondrial production in the myocardium [37], while in the present study, miRNA-27b-5p expression was downregulated and *Cxcl10* expression was upregulated; therefore, *Cxcl10* may be regulated by miRNA-27b-5p in CNI-ED. The analysis using the Circular RNA Interactome database showed that the upregulated circRNA2981, circRNA2984, circRNA3986, and circ3009 were likely to be sponges of miRNA-27b-5p. Therefore, circRNAs may cause mitochondrial dysfunction of CCSMCs by targeting *Cxcl10* through sponging miRNA-27b-5p, and ultimately leading to ED.

The mTOR signaling pathway plays a crucial role in muscle atrophy [38]. Existing evidence proves that phospholipase D (PLD), through its product phosphatidic acid combined with mTOR, plays the most useful role and ultimately stimulates the mTOR signaling pathway [39]. PLD1, as the well-known member of the PLD family, has been widely studied. Rami Jaafar found that PLD1 can simultaneously activate mTORC1 and mTORC2 which can reverse muscle atrophy and have a positive effect on muscle nutrition. In our sequencing results, the expression of *Pld1* was increased, which may indicate that PLD1 also plays a protective role in CNI-ED. We used TargetScan and miRanda to conduct miRNA-mRNA interaction analysis, looking for miRNAs that might competitively bind to PLD1, and the results showed miRNA-let-7a and miRNA-129-2-3p as candidates. We then used the Circular RNA Interactome database to look for circRNAs that were likely to sponge miRNA-let-7a and miRNA-129-2-3p. Finally we found that ciRNA351, circRNA2981, circRNA2982, circRNA2983, circRNA2986, and circRNA3009 may be involved in the regulation of *Pld1*.

There are still several shortcomings in our study. Although circRNAs are highly conserved among species, the differentially expressed circRNAs discovered via sequencing still need to be confirmed in human tissues. In addition, the relationship between circRNAs/miRNAs/mRNAs predicted by the software has not been further verified via experiments. Besides, the model we used was a CNI-ED rat model; therefore, the expression of circRNAs in patients with ED is still unknown.

## Conclusions

In a nutshell, the circRNAs with significantly different expressions were comprehensively analyzed and a circRNAs centered CERNA network was constructed. Three circRNAs with the ability to encode proteins were discovered. We identified *Ccna2, Cxcl10, Pld1, Mapk11* and *Mboat2* as potential therapeutic targets for CNI-ED. Our research on circular RNA represents a new insight into ED caused by nerve injury. However, further experiments are still needed to verify the relationships among circRNAs, miRNAs and mRNAs in the circRNA network.

## Supplementary Material

Supplemental Material

## Data Availability

The raw data supporting the conclusions of this article will be made available by the authors, without undue reservation. The sequencing results have been uploaded to the GEO database: GSE176099 study at:https://www.ncbi.nlm.nih.gov/geo/query/acc.cgi?acc=GSE176099

## Research highlights:

1. The role of circRNAs in cavernous nerve-damaging ED was assessed.

2. A ceRNA network centered on circRNA about CNI-ED is constructed.

3. The occurrence of CNI-ED is related to the regulation of energy metabolism by circRNA.

4. We identified five hub gene in the ceRNA network that play important roles in CNI-ED.
