# Supplementary material for: Whole-transcriptome analysis of rat cavernosum and identification of circRNA-miRNA-mRNA networks to investigate nerve injury erectile dysfunction pathogenesis
Source: Bioengineered. 2021 Sep 14;12(1):6516–28. doi: 10.1080/21655979.2021.1973863 (PMC8806524; doi:10.1080/21655979.2021.1973863)
Supplement: Supplemental Material [file KBIE_A_1973863_SM6315.zip › supplementary/Figure supplement.docx]

**Figure supplement**
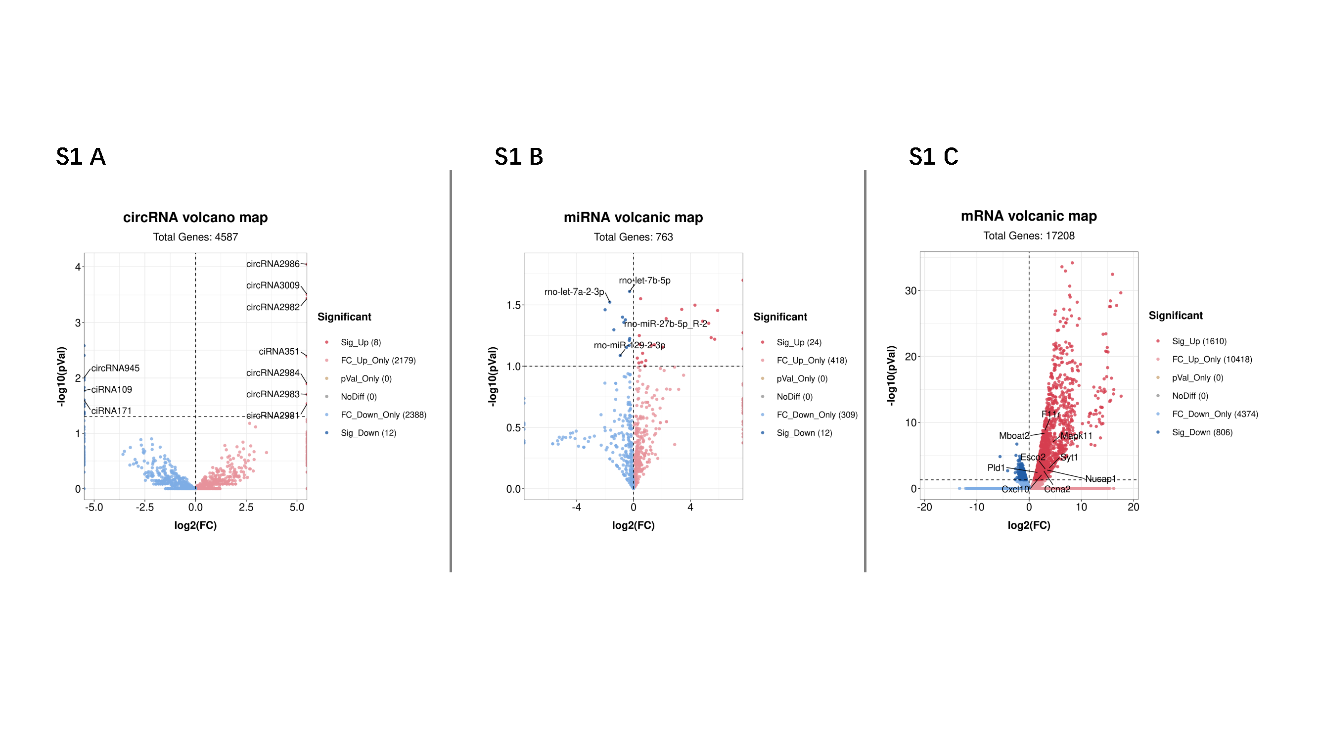
 **1:** (A-C) CircRNAs,miRNA,mRNA volcanic map in sequencing results. In the volcano plots, blue means down, red means up
